# Supplementary material for: WNT10A mutation causes ectodermal dysplasia by impairing progenitor cell proliferation and KLF4-mediated differentiation
Source: Nat Commun. 2017 Jun 7;8:15397. doi: 10.1038/ncomms15397 (PMC5467248; doi:10.1038/ncomms15397)
Supplement: Supplementary Information — Supplementary figures and supplementary table. [file ncomms15397-s1.pdf]

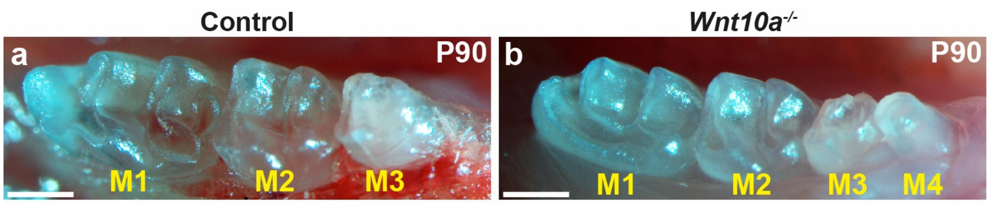

Supplementary Figure 1. *Wnt10a* mutation causes aberrant molar tooth development and formation of an ectopic M4 molar. (**a**,**b**) Smaller molar teeth with blunted cusp formation, and presence of an ectopic molar M4 in *Wnt10a*$^{-/-}$ (**b**) compared with control (**a**) mandible at P90. N$\geq$40 mutant and n$\geq$40 control mice examined. Scale bars indicate 500μm.

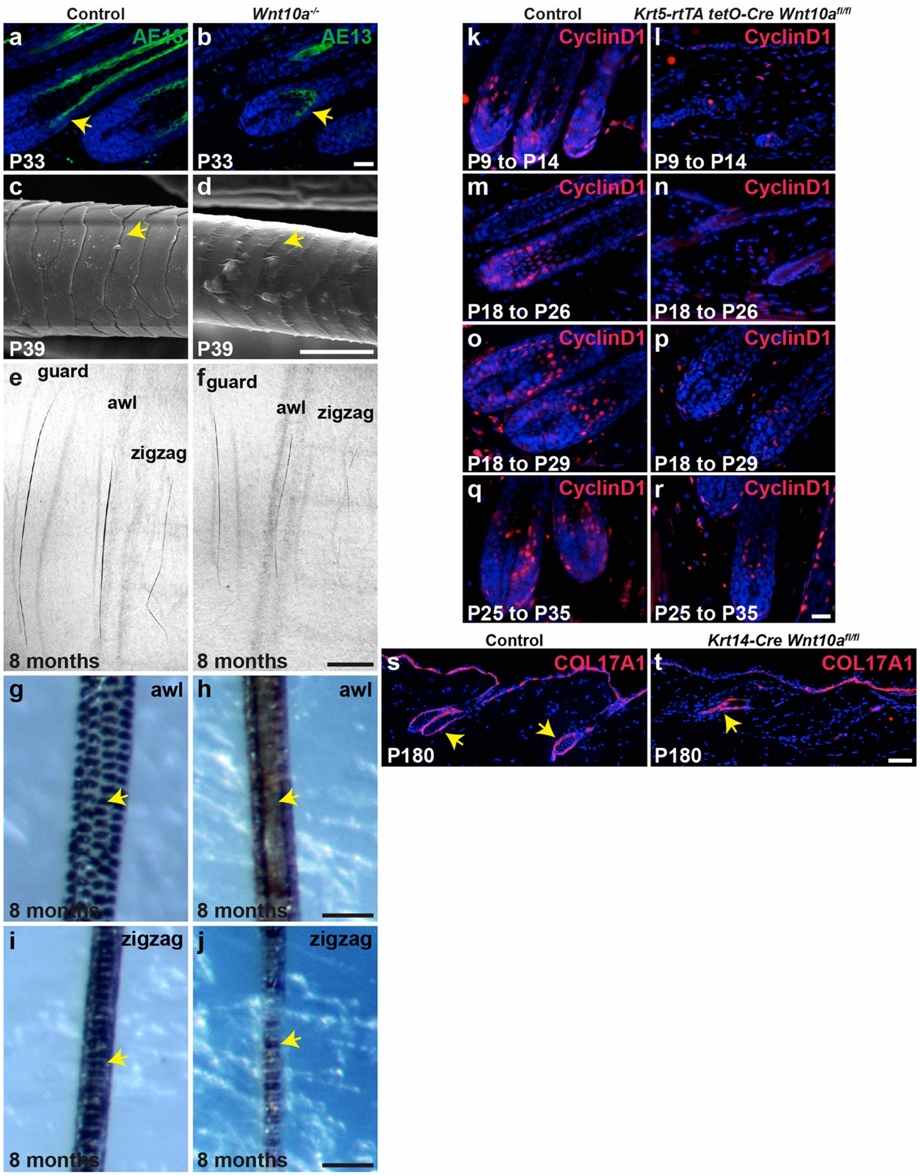

Supplementary Figure 2. Hair follicle phenotypes in *Wnt10a* mutant mice. (**a**,**b**) Immunofluorescence with antibody to hair shaft keratins (AE13, green; arrows) reveals their expression in *Wnt10a*$^{-/-}$ hair follicles at P33. (**c**,**d**) SEM shows narrowed *Wnt10a*$^{-/-}$ awl hair shaft but normal cuticle structure compared with control at P39 (arrows). The smoother appearance of the mutant hair shaft is caused by excess sebum production. (**e**-**j**) Hair shafts are shorter and narrower in *Wnt10a*$^{-/-}$ compared with control littermate mice (**e**,**f**) and have disorganized internal medulla structures (**g**-**j**; arrows). (**k**-**r**) Immunofluorescence for cyclin D1 (pink) in paraffin sectioned dorsal skin reveals its decreased expression in HF of mice with inducible loss of epithelial *Wnt10a* in embryonic anagen (**k**,**l**); following deletion before onset of the first postnatal hair cycle (**m**-**p**); or following deletion in anagen (**q**,**r**). *Krt5-rtTA tetO-Cre Wnt10a*$^{fl/fl}$ mice and littermate controls were dox-treated for the times indicated and analyzed at the end of the treatment periods. At least three controls and three mutant mice were used for each analysis. (**s**,**t**) Immunofluorescence for COL17A1 (pink) shows that its expression persists in miniaturized HF of *Krt14-Cre Wnt10a*$^{fl/fl}$ mice at P180 (arrows). Scale bars indicate 10μm (**c**,**d**), 20μm (**g**-**j**), 25μm (**a**,**b**,**k**-**r**), 50μm (**s**,**t**), or 0.65mm (**e**,**f**).

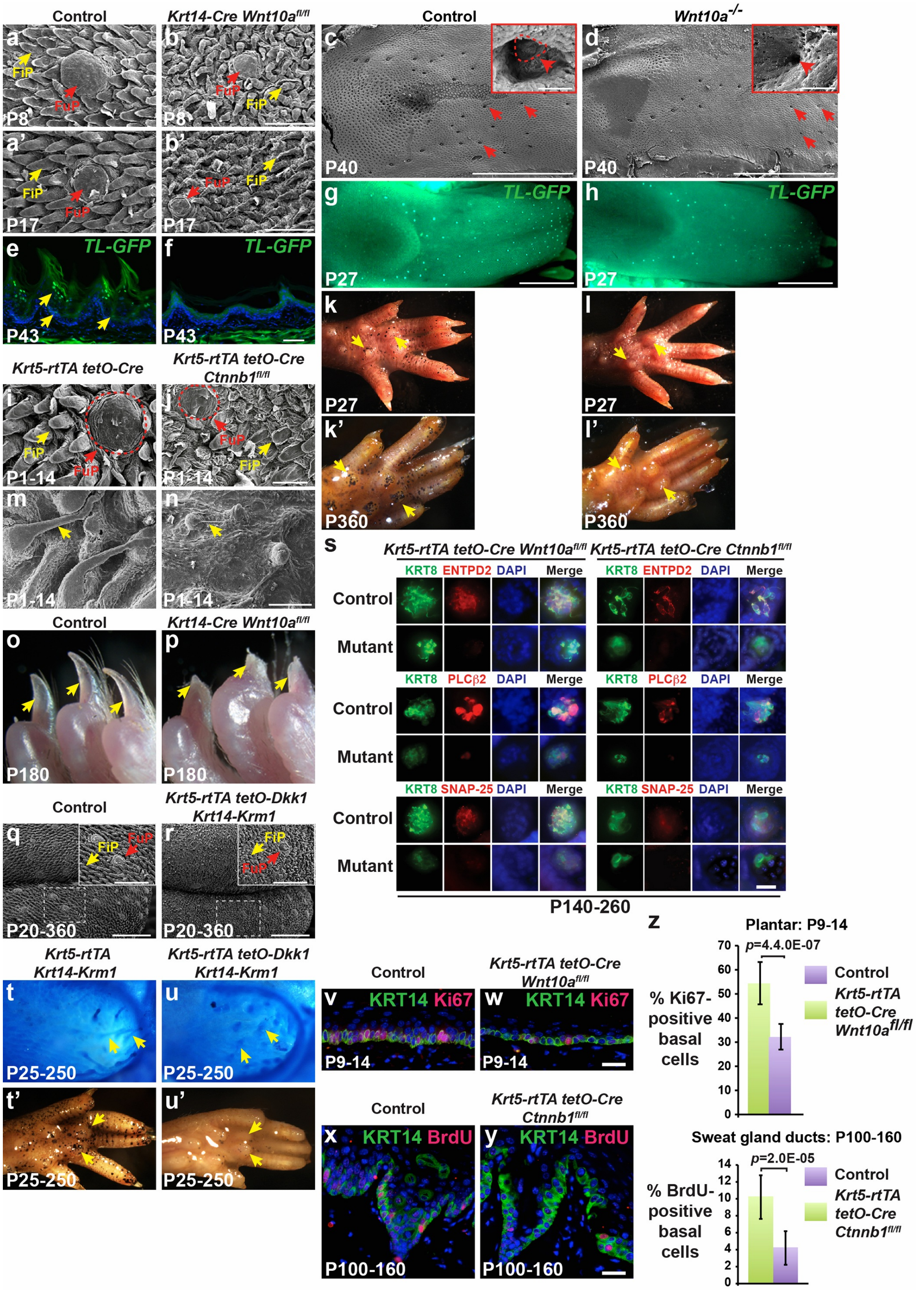

Supplementary Figure 3. ***Wnt10a*** **and β-catenin signaling are required for postnatal development and adult maintenance of multiple epithelial tissues.** (**a-b'**) SEM reveals defective fungiform (FuP, red arrows) and filiform (FiP, yellow arrows) papilla structures by P8 (**a**,**b**), and more severe defects by P17 (**a'**,**b'**) in mouse tongues

lacking epithelial *Wnt10a*. (**c,d**) SEM reveals decreased numbers of fungiform papillae and miniaturized fungiform TBs in P40 *Wnt10a*$^{-/-}$ dorsal tongue (red arrows). (**e,f**) GFP expression is decreased in P43 *K14-Cre Wnt10a*$^{fl/fl}$ *TL-GFP* dorsal tongue compared to control. (**g,h**) Reduced number of *TL-GFP*+ fungiform taste buds in P27 *Wnt10a*$^{-/-}$ *TL-GFP* compared with control tongue. (**i,j**) Epithelial β-catenin deletion from P1 causes aberrant fungiform and filiform papillae, assayed by SEM at P14. (**k-l'**) Reduced sweating in *Wnt10a*$^{-/-}$ mice compared to controls at P27 (**k,l**) and P360 (**k',l'**), assayed by starch-iodine staining (brown/purple dots, arrows). (**m,n**) Epithelial β-catenin deletion from P1 prevents sweat duct development, visualized by SEM of separated epidermis (arrows). (**o,p**) Constitutive epithelial *Wnt10a* deletion causes onychodystrophy (arrows). (**q,r**) Inducible forced *Dkk1*/*Krm1* expression from P20 causes filiform and fungiform papilla defects (arrows). (**s**) Inducible epithelial *Wnt10a* or β-catenin deletion from P140 causes decreased expression of the general TB cell marker KRT8, and specific markers of type I (ENTPD2+), type II (PLCβ2+) and type III (SNAP-25+) TB cells, assayed by whole mount immunofluorescence of separated tongue epithelium at P260. (**t-u'**) Inducible forced *Dkk1*/*Krm1* expression from P25 causes defective sweat duct structure (whole mount Nile blue staining) (**t,u**) and reduced sweating ability (starch-iodine staining) (**t',u'**, brown/purple dots) at P250. (**v,w**) Inducible *Wnt10a* deletion from P9 causes decreased proliferation in mutant plantar skin by P14. (**x,y**) Inducible deletion of epithelial β-catenin in adult life causes decreased proliferation in mutant sweat gland ducts. (**z**) Decreased proliferation in plantar epidermis and sweat ducts of *Wnt10a* and β-catenin mutants is statistically significant. Plantar epidermis: Ki67+/KRT14+ and total KRT14+ cells counted in 15 fields at 20x magnification from 3 control mice and the same for 3 mutant mice. Sweat ducts: BrdU+/KRT14+ and total KRT14+ cells counted in 10 ducts from 3 control mice and 10 ducts from 3 mutant mice. Significance was calculated with 2-tailed Student's t-test; error bars indicate s.e.m. Dox treatment periods are indicated in the bottom left of the panels. Mice were analyzed at the end of the induction time. Scale bars indicate 20μm (insets in **c,d**), 25μm (**s,v-y**), 50μm (**e,f,i,j,m,n**),100μm (**a-b'**), 250μm (insets in **q,r**), 500μm (**q,r**), or 2mm (**c,d,g,h**).

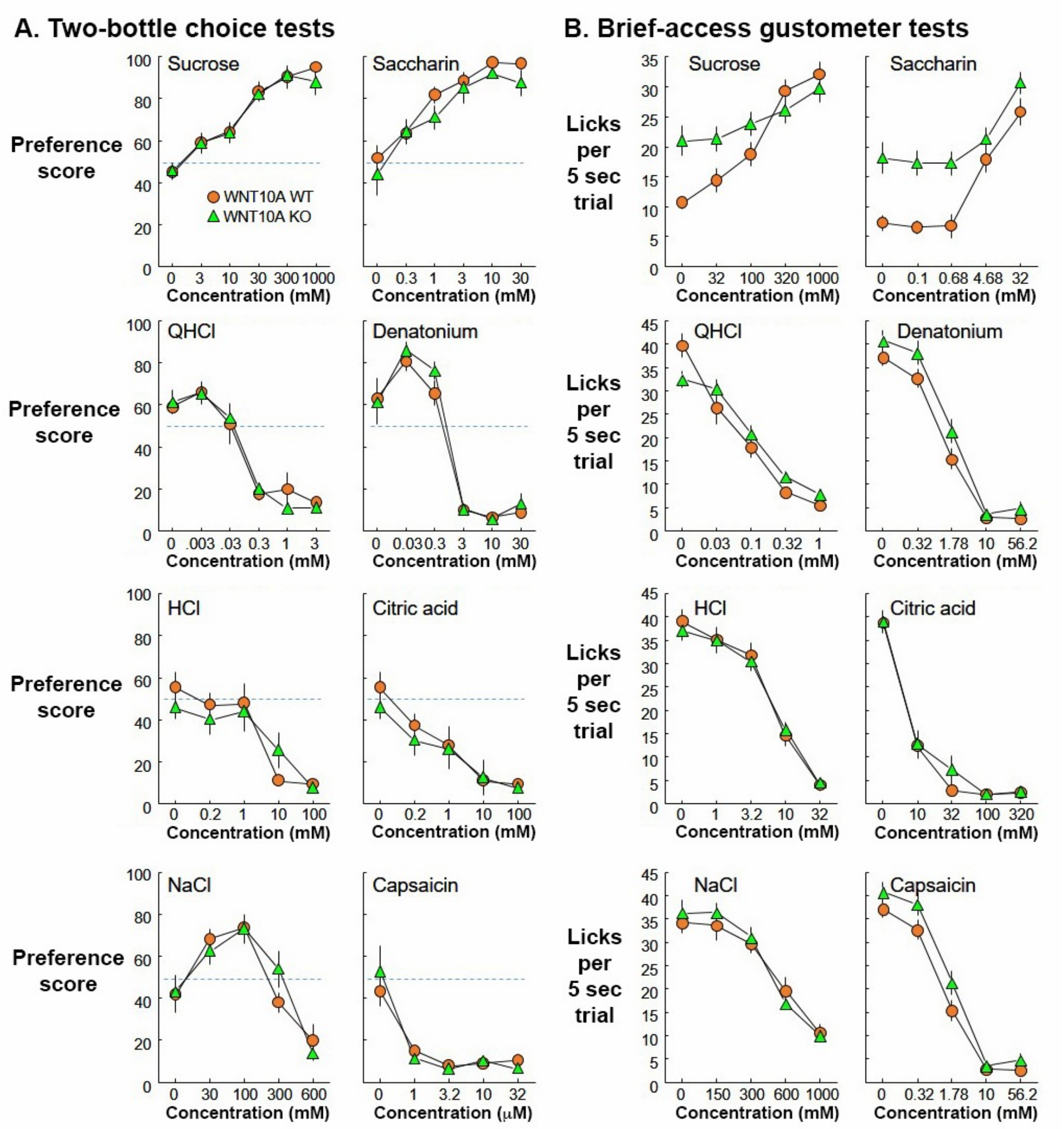

Supplementary Figure 4. *Wnt10a*$^{-/-}$ mice have normal behavioral responses to taste compounds. Results are shown for two-bottle choice tests (**A**) and brief-access gustometer tests (**B**). Values are shown as mean ± SEM for 7 – 16 mice per genotype. Two-bottle choice test values are preference scores, based on 48 hour intakes of taste solution as a proportion of the total of taste solution + water intake (x 100). Brief-access tests are licks made during 5 seconds of access to taste solution. Solution concentrations are shown as millimolar, except for capsaicin in the two-bottle choice test, which is micromolar. There were no significant differences between *Wnt10a*$^{+/+}$ and *Wnt10a*$^{-/-}$ mice with the exception that the *Wnt10a*$^{-/-}$ mice drank more water and low (most likely sub-threshold) concentrations of the sweeteners in the gustometer tests. Note that the sweeteners were tested with the mice under less severe food and water deprivation procedures than those used for the other compounds, explaining why lick rates for water differed. Post hoc least-significant difference tests were used to assess between-group differences in consumption of specific concentrations of taste solution and differences in the response of each group to individual concentrations of each taste compound. All analyses were conducted using a criterion for significance of $p<0.05$.

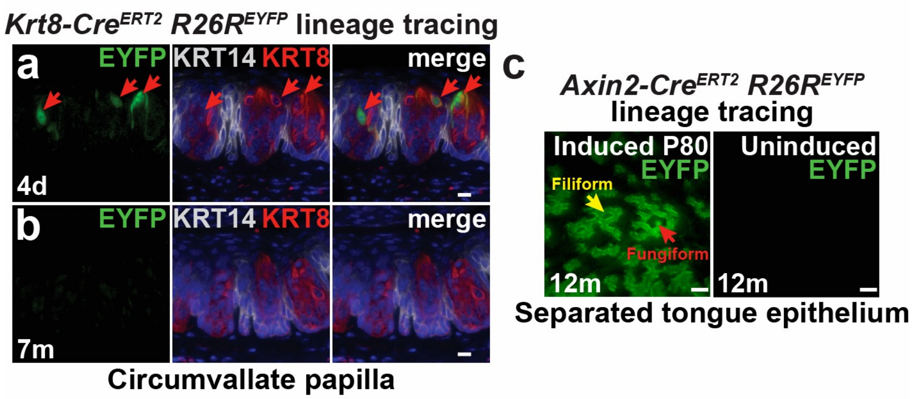

Supplementary Figure 5. Lineage tracing of *Krt8*+ cells in circumvallate papilla and *Axin2*+ cells in dorsal tongue epithelium. (**a**,**b**) Lineage tracing of *Krt8*+ differentiated taste bud cells in circumvallate papillae. Adult *Krt8-Cre^{ERT2} R26R^{EYFP}* mice were i.p. injected with tamoxifen on 2 successive days and analyzed after 4 days (**a**) or 7 months (**b**) for expression of EYFP, KRT14 and KRT8. At 4 days, KRT8+ differentiated taste bud cells were EYFP+ (arrows). EYFP label was lost by 7 months, indicating that these cells cannot self-renew. (**c**) *Axin2-Cre$^{ERT2/tdT}$ R26R^{EYFP}* mice were tamoxifen treated at P81 and P82 or left untreated. Whole mount imaging of the underside of separated tongue epithelium after 12 months revealed EYFP+ cells in fungiform (red arrow) and filiform (yellow arrow) papillae in tamoxifen-treated mice, but not in un-induced mice. At least 3 mice were analyzed for each tracing condition. Scale bars indicate 10μm (**a**,**b**) or 100μm (**c**).

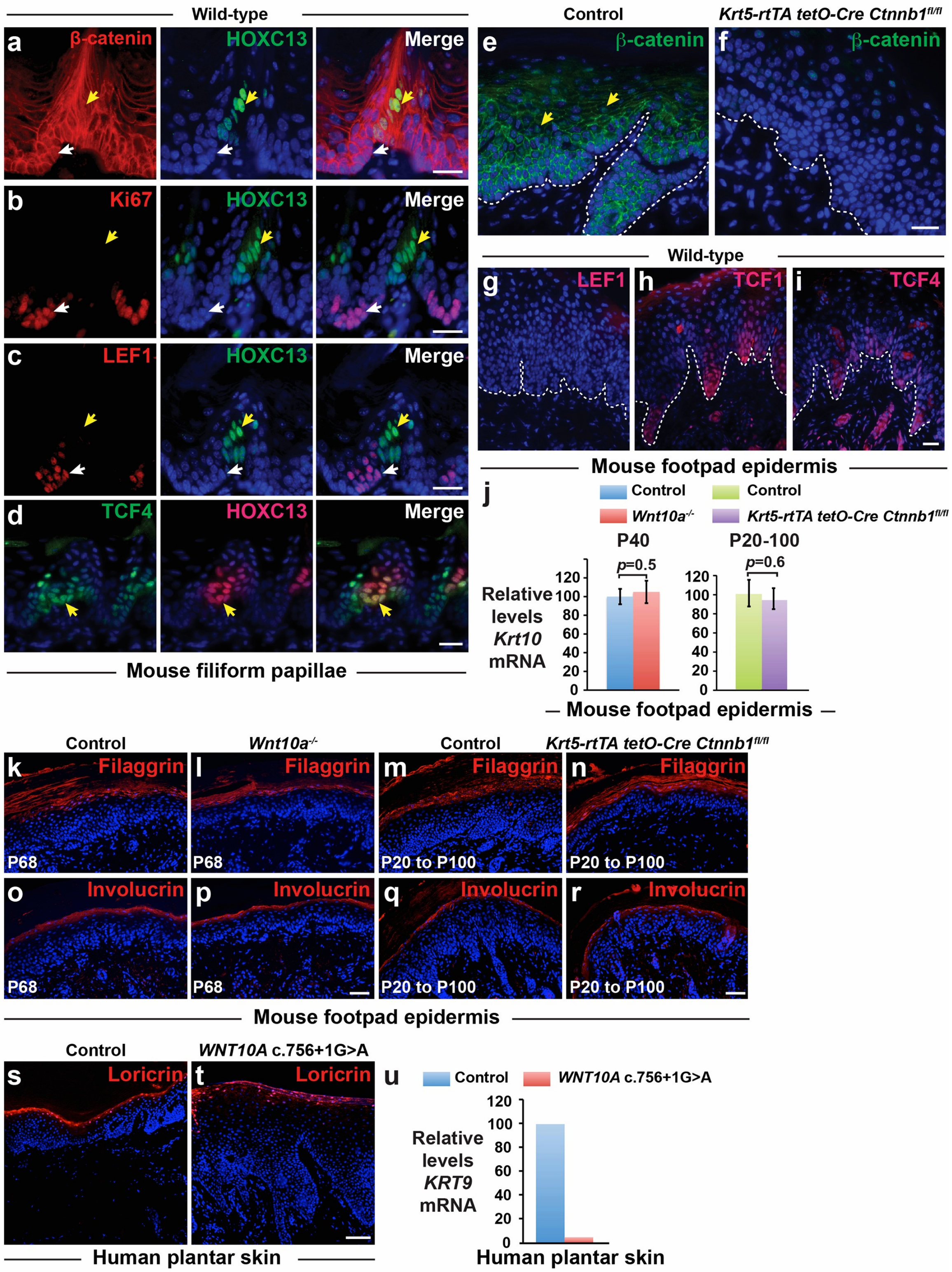

Supplementary Figure 6. Wnt signaling pathway components localize to progenitor and differentiating cells in filiform papillae and footpad epidermis, and control region-specific but not generalized differentiation programs. (**a-d**) Co-immunostaining of dorsal tongue for HOXC13 (**a-c**, green; **d**, red) and β-catenin (**a**, red), Ki67 (**b**, red), LEF1 (**c**, red), or TCF4 (**d**, green). HOXC13 expression overlaps with

nuclear β-catenin (**a**) and TCF4 (**d**). White arrows indicate cells that are HOXC13- but are positive for nuclear β-catenin (**a**), Ki67 (**b**), or LEF1 (**c**). Yellow arrows indicate differentiating HOXC13+ cells that are positive for nuclear β-catenin (**a**) and TCF4 (**d**) and are LEF1- (**c**). (**e**) β-catenin immunofluorescence of footpad skin from P45 *Krt5-rtTA tetO-Cre Ctnnb1*$^{fl/fl}$ mutants and littermate controls induced from P1. Yellow arrows indicate nuclear β-catenin. (**g-i**) Immunofluorescence of wild-type footpad skin for LEF1 (**g**), TCF1 (**h**), or TCF4 (**i**). (**j**) qPCR of *Krt10* mRNA in mouse footpad epidermis shows no significant differences between P40 *Wnt10a*$^{-/-}$ mutants, mice with epithelial β-catenin deletion induced from P20 and analyzed at P100, and littermate controls. *N*=6 control and 6 mutant mice at P40 and n=4 control and 4 mutant mice at P20-100; 3 technical replicates per sample; normalized to β-actin. Significance was calculated with 2-tailed Student's t-test. Error bars indicate s.e.m. (**k-r**) Immunofluorescence (red) for filaggrin (**k-n**) or involucrin (**o-r**) in footpad epidermis reveals unchanged expression in *Wnt10a*$^{-/-}$ compared with control mice (**k,l,o,p**) or following inducible deletion of β-catenin from P20 (analyzed at P100) (**m,n,q,r**). (**s-u**) Plantar skin from a homozygous *WNT10A c.756+1G>A* human patient compared to a similarly aged sex-matched control shows similar expression of loricrin, analyzed by immunofluorescence (**s,t**; red) and decreased *KRT9* mRNA in the patient sample, analyzed by qPCR (**u**). For qPCR of human samples, mRNA was extracted from paraffin sections and *KRT9* mRNA levels were normalized against *KRT10*. Three technical replicates were performed. Scale bars indicate 25μm (**a-i**), 50μm (**k-r**) or 100μm (**s,t**).

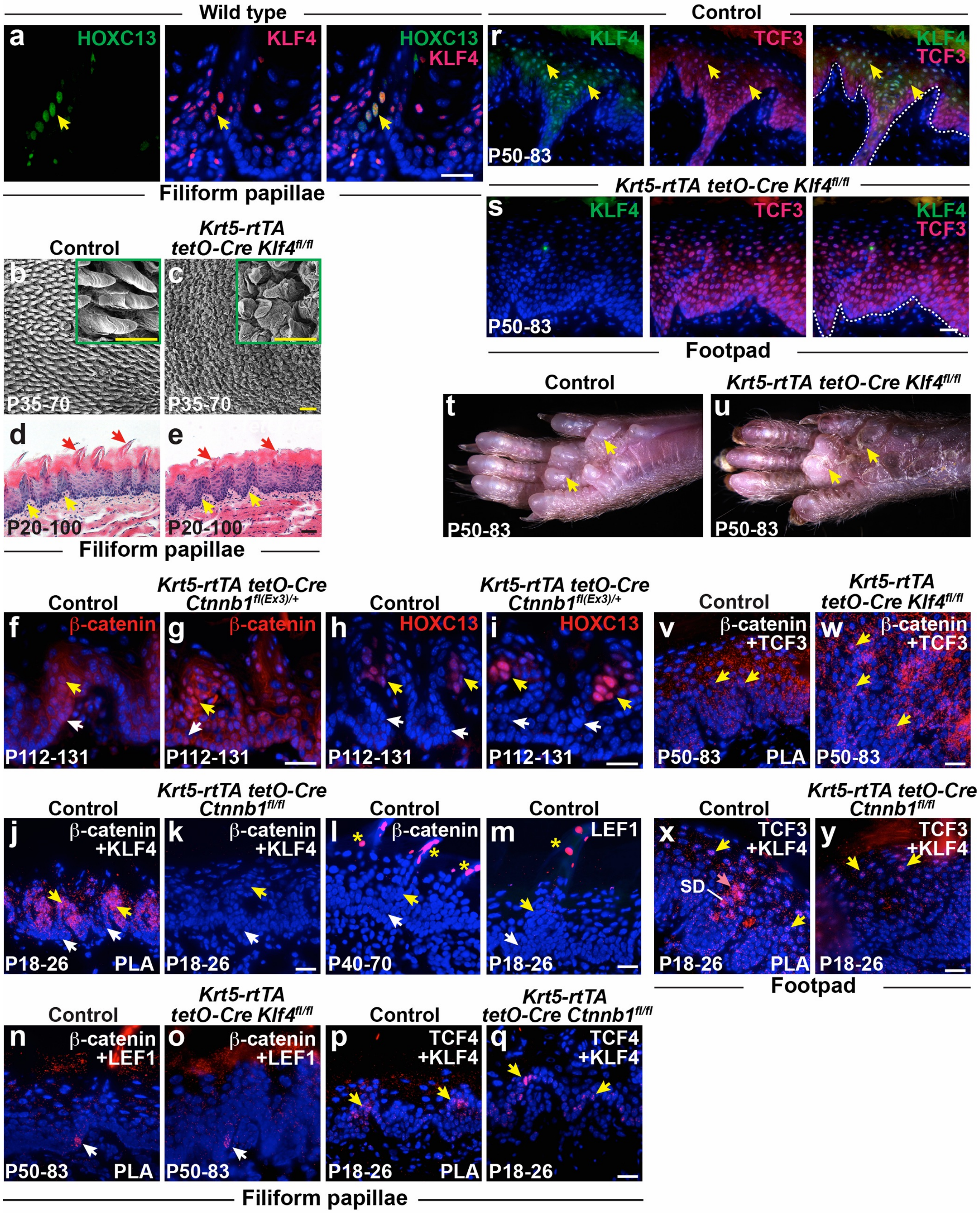

Supplementary Figure 7. β-catenin interacts differentially with TCF/LEF1 and KLF4 proteins to control proliferation and differentiation of specialized epithelia. (**a**) Expression of HOXC13 (green) and KLF4 (pink) overlaps in differentiating tongue filiform papillae. (**b**,**c**) Induced deletion of epithelial *Klf4* from P35 (**b**,**c**) or P20 (**d**,**e**) causes filiform papilla structural defects, analyzed by SEM at P70 (**b**,**c**) and H&E staining at P100 (**d**,**e**). Yellow arrows indicate basal cells; red arrows indicate horny tip structures, which are defective in the mutants. (**f-i**) Nuclear localized β-catenin (**f**,**g**) and HOXC13

(**h**,**i**) are elevated in filiform papillae of adult mice with induced mutation of epithelial β-catenin to a stabilized form (yellow arrows), but mutant basal cells do not display ectopic HOXC13 expression (**h,i**, white arrows). (**j-q**) PLA in dorsal tongue sections. Antibodies to β-catenin and KLF4 yield positive signals in differentiating filiform papillae cells in control tissue (**j**) and absence of signal following inducible epithelial β-catenin deletion (**k**). PLAs with single antibodies to β-catenin (**l**) or LEF1 (**m**) yield no specific signal. Interaction of β-catenin and LEF1 is unaffected by inducible *Klf4* deletion (**n,o**); interaction of TCF4 and KLF4 is unaffected by inducible β-catenin deletion (**p,q**). White arrows indicate proliferating basal cells; yellow arrows indicate differentiating cells. (**r,s**) KLF4 and TCF3 expression overlap in suprabasal footpad epidermis (**r**, yellow arrows); TCF3 expression is retained in suprabasal cells following inducible *Klf4* deletion (**s**). (**t,u**) Inducible epithelial *Klf4* mutant mice display scaling and hyperpigmentation of footpad skin (yellow arrows). (**v-y**) PLA in sectioned footpads. β-catenin and TCF3 interact in basal and suprabasal epidermis (**v**); this is unaffected by inducible deletion of *Klf4* (**w**). TCF3 and KLF4 interact in suprabasal epidermis (**x**); this is unaffected by inducible deletion of β-catenin (**y**) (yellow arrows). Strong signals in the control localize to sweat duct (SD) cells (**x**, pink arrow). For inducible deletion, mutant and littermate control mice were induced between the stages indicated in the lower left of each panel and tissues were harvested at the end of the induction time. At least three control and three mutant mice were analyzed in each experiment. Scale bars indicate 25μm (**a,f-s,v-y**), 50μm (**d,e**; insets in **b,c**), or 100μm (**b,c**).

Supplementary Table 1. Sequences of primers used for ChIP and qPCR.

**qPCR Primers**	
mKrt9-RT-5'	5'GGTAGCTATGGTGGAGGAAATAG3'
mKrt9-RT-3'	5'GTCCGGTGGAGAAAGTGAAT3'
mKrt10-RT-5'	5'CTCACCCTGACAACTGACAAT3'
mKrt10-RT-3'	5'GGGTCACCTCATTCTCGTATTT3'
actin-RT-5'	5'AGATTACTGCTCTGGCTCCTA3'
actin-RT-3'	5'CTGCTTGCTGATCCACATCT3'
Hoxc13-RT-5'	5'AGTCAGGTGTACTGCTCCA3'
Hoxc13-RT-3'	5'CTTCAGCTGCACCTTGGTAT3'
HKRT9-RT-5'	5'CATCCCATTCCTAGTCTTCTTCC3'
HKRT9-RT-3'	5'GGGCAGGAGAAGTTTACAGAG3'
HKRT10-RT-5'	5'GTGGGCGAGTCTTCATCTAAG3'
HKRT10-RT-3	5'GGCGCCACCTCTTCAATAA3'
HWNT10A-Exon1-2-RT5'	5'GTGCTCCTGTTCTTCCTACTG3'
HWNT10A-Exon1-2-RT3'	5'CACACTGTGTTGGCATTGAG3'
HWNT10A-Exon2-3-RT5'	5'TCCCATCTTCAGCAGAGGTTTC3'
HWNT10A-Exon2-3-RT3'	5'AGCCACAGGCCTTCAGTTT3'
HWNT10A-Exon3-4-RT5'	5'TTTCTAAGGACTTTCTGGACTC3'
HWNT10A-Exon3-4-RT3'	5'CCGCATGTTCTCCATCACT3'
HWNT10A-Exon4-RT5'	5'TTCGTGGTCTGCGAAGAGT3'
HWNT10A-Exon4-RT3'	5'CCAAGACCGTAAGCCTCAGA3'
**ChIP Primers**	
Krt9-ChIP-5'	5'GGGAGGAGACTCAGAGATCAAA3'
Krt9-ChIP-3'	5'TGAAGGTAACAGCCAGATTGC3'
